# Shaped by context: Evolutionary trajectories of desiccation tolerance in land plants

**DOI:** 10.1002/ajb2.70180

**Published:** 2026-03-24

**Authors:** Rose A. Marks, R. Shawn Abrahams, Jenna T. B. Ekwealor

**Affiliations:** ^1^ Department of Plant Biology University of Illinois at Urbana‐Champaign Urbana IL USA; ^2^ Department of Biology San Francisco State University San Francisco CA USA

**Keywords:** anhydrobiosis, desiccation tolerance, evolutionary trajectories, gametophyte, macroevolution, modularity, poikilohydry, resurrection plants, sporophyte, systems biology

## Abstract

Desiccation tolerance (DT), the ability to survive near‐complete cellular dehydration, is widespread in diaspores but rare in the vegetative tissues of land plants. The patchy and punctuated phylogenetic distribution of vegetative desiccation tolerance (VDT) suggests that the trait is both ancient and recurrent, yet the evolutionary trajectories remain unresolved. Here, we synthesize evidence across land plants to propose a framework for the evolution of VDT in embryophytes. We build on the current understanding of VDT as an ancestral trait, present in the gametophyte of early land plants. The transition to sporophyte dominance and resulting homiohydry in vascular plants coincides with the widespread loss of VDT, likely driven by relaxed selection for VDT, coupled with new structural constraints and anatomical innovations that facilitated water acquisition, transport, and retention. The core molecular modules of DT were retained in the diaspores of most land plants, where they served as evolutionary refugia for the essential building blocks of the trait. Some species later reestablished VDT by co‐opting deeply conserved diaspore modules and evolving key anatomical innovations to support them. We argue that such reestablishments of VDT are dependent on both anatomical predispositions as well as exposure to key selective pressures and ecological filters. We conclude that VDT is not a simple presence–absence trait, but rather a modular system, subject to anatomical constraints and contingent on the ecological context. Ultimately, we suggest that VDT serves as an elegant example of how complex traits emerge, persist, and shift across time.

Water is fundamental to the structure and function of life on Earth. The vast majority of biological processes occur in an aqueous environment, and even brief periods of cellular dehydration are lethal to most cells and organisms. However, there is a specialized subset of life that can survive the near‐complete loss of cellular water. This ability, known as desiccation tolerance (DT, also desiccation‐tolerant), allows cells, tissues, and whole organisms to dry to the point that metabolism effectively ceases (Oliver et al., 2020; Gechev et al., 2021; Marks et al., 2025). Remarkably, DT cells are protected in this dry state, their function preserved, and normal metabolism is reinitiated upon rehydration, often within just a few short hours of rewetting (Bewley, 1979; Crowe et al., 1992; Marks et al., 2025). While most plant and animal tissues are not DT, this ability is present in specific life stages and cell types across a broad array of taxa including bacteria, fungi, animals, and plants (Marks et al., 2025). More than just a biological curiosity, DT plays a critical role in the survival, reproduction, dispersal, and establishment of many species (Koster and Leopold, 1988; Alpert, 2005; Oliver et al., 2005; Marks et al., 2024b), and its evolutionary history offers a unique perspective on how complex traits persist, shift, and reemerge across time.

Most land plants restrict DT to their diaspores (e.g., seeds and spores), while only a small number of “resurrection” plants express the trait in vegetative tissues. Here, we use the terminology of vegetative desiccation tolerance (VDT) to describe desiccation tolerance in vegetative life stages, but focus on the evolutionary trajectories of desiccation tolerance in photosynthetic tissues, which we argue are the primary targets of selection shaping VDT. Nonphotosynthetic tissues (e.g., roots and rhizoids) remain comparatively understudied, creating a gap in our understanding of whether they evolved VDT along distinct paths. Desiccation tolerance is a survival mechanism that enables life in water‐limited environments and microhabitats, and resurrection plants are found abundantly in seemingly inhospitable and extreme environments (Alpert, 2005; Porembski et al., 2021). On a cellular level, DT involves adaptations that both protect against, and repair from, the damage caused by desiccation (Gaff and Hallam, 1974; Gaff, 1997; Alpert, 2000; Oliver et al., 2005; Marks et al., 2025). These include increased mechanical flexibility, cellular stabilization via vitrification, synthesis of protective compounds, and efficient detoxifying mechanisms (Ghasempour et al., 1998; Farrant et al., 2007; Smolikova et al., 2020). These core mechanisms are broadly shared across DT organisms of all types (Marks et al., 2025), but additional protective mechanisms are required for VDT, and there is evidence of lineage‐specific nuances in VDT (Sherwin and Farrant, 1996; Farrant, 2000; Oliver et al., 2000; Marks et al., 2024b), highlighting the complexity of this emergent trait.

Streptophytic algae and ancestral land plants likely colonized terrestrial habitats by evolving VDT, a key adaptation that allowed them to persist through frequent wet–dry cycles (Oliver et al., 2005; Gaff and Oliver, 2013). Today, VDT is common among extant bryophytes, intermediate in extant pteridophytes (Pittermann et al., 2013), absent in gymnosperms, and extremely limited in extant angiosperms (Oliver et al., 2000; Marks et al., 2021a) (Figure 1). The punctuated and patchy phylogenetic distribution of VDT suggests that the trait is both ancient and recurrent (Gaff and Oliver, 2013), but the precise evolutionary histories of VDT are unclear. Understanding the contexts in which VDT originally arose, persisted, was lost, and subsequently reemerged is important for reconstructing plant evolutionary history, and for identifying the anatomical, molecular, and ecological conditions that support and constrain this unique survival mechanism.

**Figure 1:**
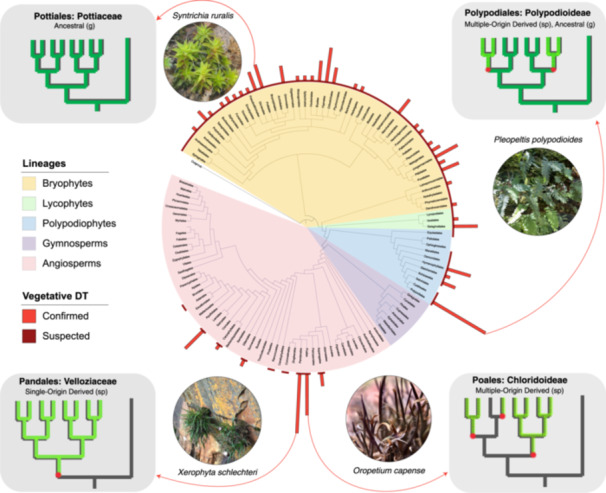
Phylogenetic distribution of vegetative desiccation tolerance (VDT) in land plants (Embryophyta) at the level of order. The distribution of “confirmed VDT” (light red bars) is shown for the number of VDT species in each order, compiled from Marks et al. (2021a) and updated with current literature (Appendix S1). Suspected VDT (dark red indicator band) is inferred by the authors based on the literature. Cartoon phylogenies shown for example clades representing different evolutionary trajectories to VDT. The color of the branches indicates the presence of VDT, with dark green indicating ancestral VDT, light green indicating derived VDT, and red stars indicating independent gains of VDT. The tissue in which VDT is expressed is indicated in the subtitles with (g) and (sp) referring to gametophyte or sporophyte tissues, respectively. Representative photos of species within each of the example families are shown in their native habitat. The base tree was drawn using the One Tree of Life data set (ott3.7.2) as a backbone with missing clades added without comment on branching order. Photos taken by the authors.

Understanding the history of VDT calls for attention not only to the presence or absence of the trait, but to the trajectory of the trait—its movement through evolutionary space shaped by context, constraint, and contingency (Edwards, 2019). Here, trajectory refers not to a fixed path, but to a flexible course influenced by preconditions and redirected over time by internal and external forces. In evolution, such forces may include anatomical constraints, gene regulatory architecture, developmental constraints, and ecological filters (Mazer et al., 2022). For example, the evolutionary trajectories of C_4_ and CAM photosynthesis show that outcomes depend not only on which traits are most adaptive, but also on which trajectories are accessible, given context and constraint (Edwards, 2019). The repeated, convergent origins of VDT, much like C_4_ and CAM photosynthetic pathways, are shaped by structural preconditions that constrain or permit the stepwise assembly of the emergent phenotype.

In this viewpoint, we explore how DT varies across tissues, life stages, and clades of land plants to propose an evolutionary framework for how it has evolved, has been maintained, lost, and repurposed for different contexts. We begin with the current understanding that DT is an ancient trait—likely present in the ancestor of land plants and the gametophytic life stages of early land plants—and that despite being absent from the vegetative tissues of most extant sporophytes, the core mechanisms have been retained in spores, seeds, and other reproductive tissues as a diaspore DT (DDT) functional unit (Gaff and Oliver, 2013; Costa et al., 2017b). Building on this understanding, we propose that not only has DDT served as an evolutionary refugia for the core molecular modules of DT, but that only when specialized anatomical modules are present and given the appropriate ecological filters, could the VDT functional unit become reestablished in vegetative tissues of extant sporophytes. We argue that the core molecular modules of DT are deeply conserved, but that the emergence of VDT is context dependent, convergent, and shaped by the interplay of anatomy, biochemistry, and natural selection. We employ concepts from systems biology to describe DT as two distinct functional units that selection acts on independently that are built from multiple underlying modules, which are defined as either molecular (i.e., biochemical) or anatomical (i.e., developmental) (Figure 2). We view the recurrent emergence of VDT across land plants not simply as repeated convergent evolution, but as context‐limited reestablishments of a deeply conserved trait that was never entirely lost.

**Figure 2:**
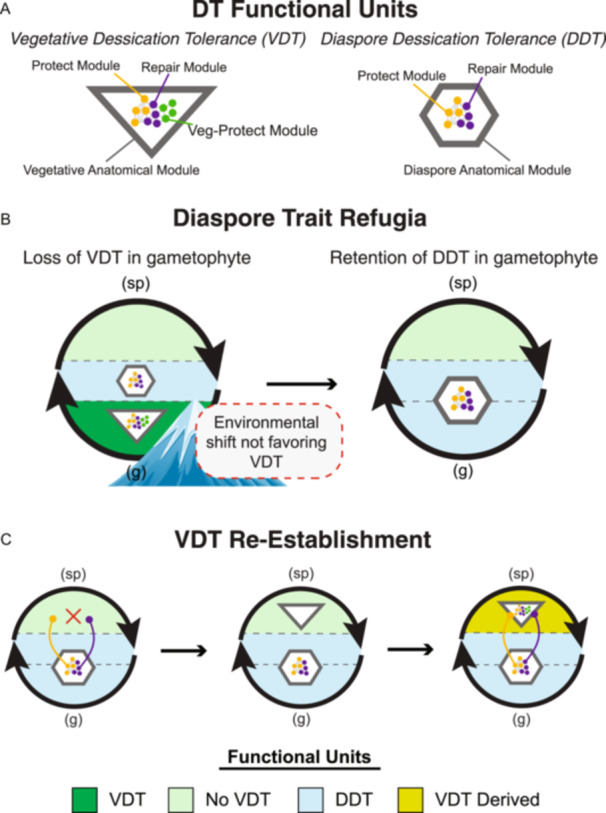
Evolutionary trait refugium model for desiccation tolerance (DT). (A) DT functional units show that both vegetative desiccation tolerance (VDT) and diaspore desiccation tolerance (DDT) share core protect and repair molecular modules, but only VDT includes a vegetative‐protect module and requires a vegetative anatomical module, while DDT uses a diaspore anatomical module. (B) Diaspore trait refugia demonstrates that during an environmental shift that disfavors VDT, the diaspore's DDT acts as a trait refugium, retaining the essential DT core modules even if VDT is lost in the gametophyte (g; sp, sporophyte). (C) VDT reestablishment illustrates that for VDT to be regained in the vegetative life stage, the core DDT modules must be co‐opted following the convergent evolution of the required vegetative anatomical module; this anatomical structure then allows for the subsequent co‐option or convergent evolution of the vegetative‐protect module, thus restoring full VDT function.

## THE ANCESTRAL STATE IN LAND PLANTS: DESICCATION TOLERANCE IN GAMETOPHYTES

Bryophytes provide comparative insight into how VDT may have been expressed by the ancestor of embryophyta. The most recent common ancestor of land plants is thought to have had a body plan similar to that of extant bryophytes, including a dominant DT gametophyte stage, which is consistent with the view that VDT was ancestral to land plants and has been maintained in extant bryophytes over evolutionary time (Oliver et al., 2000; Gaff and Oliver, 2013; de Vries and Archibald, 2018; Bowles et al., 2023). Most extant bryophyte species are suspected to have VDT, although only 1% are confirmed (Proctor and Pence, 2002; Proctor et al., 2007; Wood, 2007; Figure 1). The true frequency of VDT in extant bryophytes is poorly documented and largely inferred, requiring additional field observations and experimental tests to fully resolve its frequency and distribution (Proctor et al., 2007; Wood, 2007; Marks et al., 2021a). Still, VDT is considered common in the dominant haploid gametophyte life stage of extant bryophytes, and in many cases, bryophytes have both VDT in their gametophyte tissues (e.g., moss shoots and liverwort thalli) and DDT in propagule tissues such as gemmae and spores (Proctor et al., 2007; Wood, 2007; Coe et al., 2021; Peñaloza‐Bojacá et al., 2023). Bryophyte sporophytes are also sometimes DT, but because they are generally short‐lived, only weakly photosynthetic (Jauregui‐Lazo et al., 2023), and largely homiohydric (Proctor et al., 2007; Goffinet and Shaw 2008; Kubásek et al., 2021; Duckett and Pressel, 2018, 2022) their DT is more similar to DDT (Proctor et al., 2007; Kubásek et al., 2021; Duckett and Pressel, 2018, 2022). Despite being broadly underrepresented in the literature (Marks et al., 2021b, 2023), some bryophyte model clades for DT have emerged, most notably the mosses *Syntrichia ruralis* (previously *Tortula ruralis*), *Bryum argenteum*, and *Ceratodon purpureus*.

Bryophyte gametophytes possess anatomical and morphological traits that both facilitate DT and subject them to strong selection for its maintenance (Figure 2). Their thin tissues (many moss leaves are only one cell thick) and the absence of a thick waxy cuticle allow for fluid conformational changes during drying (Cruz de Carvalho et al., 2019; Jauregui‐Lazo et al., 2023). The lack of stomata and complex vascular systems also result in rapid equilibration with ambient moisture and frequent desiccation–rehydration cycles (Merced and Renzaglia, 2017; Morales‐Sánchez et al., 2022; Duckett et al., 2024). This poikilohydric habit, in which tissue hydration closely tracks environmental humidity, is a defining feature of many bryophytes (Proctor et al., 2007) and places them under strong selection for DT, such that even in mesic and tropical habitats, VDT is common in bryophytes. Coupled with efficient metabolic and biochemical adaptations for protection and repair during desiccation and rehydration (Oliver et al., 2005), these traits help explain the prevalence of both VDT and DDT in extant bryophytes.

The ability of bryophytes to tolerate desiccation is supported by protective and reparative molecular modules that operate before, during, and after drying. Bryophytes can survive both rapid and prolonged desiccation (Proctor et al., 2007; Coe et al., 2025) through a combination of preemptive protection during drying and responsive repair processes activated during rehydration (Oliver et al., 2000, 2005; Proctor et al., 2007). Protective mechanisms include constitutively expressed proteins such as late embryogenesis abundant (LEA) proteins and other intrinsically disordered proteins (IDPs) (Oliver et al., 2005) and the accumulation of nonreducing sugars such as sucrose, trehalose, and raffinose (Oliver et al., 2020). Photoprotective pigments limit light‐induced damage, while efficient antioxidant systems detoxify the cellular environment during drying (Proctor et al., 2007). Upon rehydration, a suite of repair pathways are activated, including protein recycling and remodeling, membrane repair, and continued detoxification (Oliver et al., 2005; Proctor et al., 2007). The response can be thought of as a two‐phase strategy: elevated baseline protection before and during desiccation, followed by damage repair and recovery during rehydration (Oliver et al., 2005). Many bryophytes exhibit constitutive VDT, both in the sense that they maintain high levels of protective macromolecules (e.g., transcripts, proteins, metabolites) (Oliver et al., 2000) and in that they survive desiccation events regardless of the intensity (usually measured as speed). However, there are exceptions. Some bryophytes from moist microhabitats (e.g., forest understories) express VDT only when dehydration is slow (Stark, 2017), preceded by mild water stress (Beckett et al., 2005), or induced by ABA treatment (Yotsui et al., 2016). Conversely, even highly DT mosses may lose tolerance when grown in continuously hydrated conditions (Schonbeck and Bewley, 1981; Stark et al., 2017). These patterns raise important questions about the extent and nature of phenotypic plasticity in bryophyte DT.

More extensive quantification of DT across bryophytes is required to fully reconstruct its ancestral state, particularly as our models of land plant evolution continue to shift. Current evidence suggests that VDT was a foundational trait for the colonization of land and that poikilohydric gametophytic tissues were the original context in which it evolved and was maintained (Oliver et al., 2000, 2005; Gaff and Oliver, 2013; VanBuren et al., 2019). We suggest that VDT involves a multipart coordinated response with distinct protective, photoprotective, and reparative molecular modules. While bryophytes may give us insight into the nuances of VDT as expressed by the ancestor of embryophyta, the consequences of the transition to sporophyte dominant life histories provide another window into the evolutionary story of VDT.

## SPOROPHYTE DOMINANCE AND LOSS OF VEGETATIVE DESICCATION TOLERANCE

**Figure 3:**
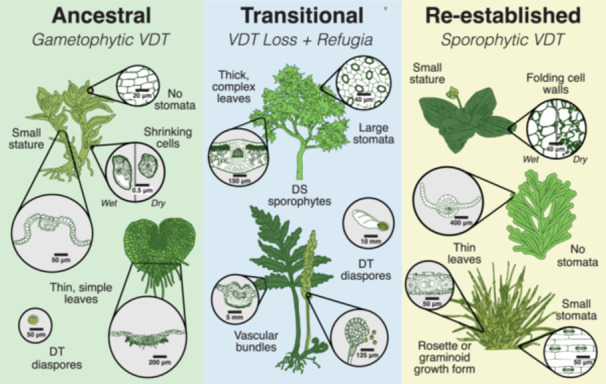
Anatomical contexts for desiccation tolerance (DT) in land plants. Ancestral land plants likely had DT spores and gametophytes with thin leaves, minimal cuticle, and no stomata, promoting a poikilohydric habit with frequent desiccation. The evolution of vasculature and other complex anatomical traits, including thick leaves with stomata, hindered DT in vegetative tissues, leading to the widespread loss of VDT and a high proportion of desiccation‐sensitive sporophytes. However, DT persisted in most reproductive propagules (diaspores) such as spores, pollen, and seeds (transitional context; middle panel). In some lineages, the core molecular modules for DT retained in diaspores were later reestablished in vegetative sporophytic tissues when ecological and anatomical conditions permitted, for example, in species with thin leaves, narrow vascular bundles, and small stomata (reestablishment; right panel). Circles represent magnified traits; dashed lines indicate leaf or thallus cross sections in magnified views.

The evolutionary transition from gametophyte to sporophyte dominance marks one of the most significant shifts in the evolutionary history of land plants (Silvestro et al., 2015). As tracheophytes emerged, sporophytes became the dominant, long‐lived, and photosynthetically active phase of the plant's life cycle. This shift coincided with major anatomical innovations including vascular tissue, lignified cell walls, and a thick waxy cuticle (Figure 3), which facilitated larger size via mechanical support, as well as internal water regulation and transport (Silvestro et al., 2015; Lu et al., 2020; Donoghue et al., 2021). These features enabled homoiohydry, reducing the frequency and severity of dehydration events experienced by those plant tissues, thereby relaxing selective pressure for VDT. Indeed, the sporophyte‐dominant life cycle is associated with the evolution of numerous dehydration avoidance mechanisms, such as deep rooting, responsive stomatal activity, and increased water storage capacity (Lu et al., 2020; McAdam et al., 2021), all of which minimize exposure to desiccation. The resulting relaxation of selective pressure for DT in vascularized vegetative tissues coincides with the widespread loss of VDT in most tracheophytes (Figures 1 and 2).

Pteridophytes (lycophytes and polypodiophytes; ferns) provide a useful intermediate reference point for understanding the relationship between DT and the transition to sporophyte dominance. Most pteridophytes have dominant sporophytic life stages as well as free‐living, anatomically simple, and poikilohydric gametophytes (Watkins et al., 2007), which share many features with bryophyte gametophytes (Figure 3). Like bryophytes, these pteridophyte gametophytes are exposed to frequent water availability fluctuations and selective pressure to maintain VDT. Current evidence suggests that a high proportion of pteridophyte gametophytes have VDT (Watkins et al., 2007; Pittermann et al., 2013), although undersampling and missing data are an issue in pteridophytes as well. The suspected high frequency of VDT in fern gametophytes reinforces the notion that gametophytic VDT is an ancestral trait maintained across millions of years.

In contrast, the structural complexity of tracheophyte sporophytes (e.g., thicker tissues, more rigid cell walls, and in some cases larger leaf area) imposes physical constraints on desiccation and rehydration (Figure 3). These anatomical features constrain the expression of DT in sporophytic tissues by reducing flexibility and confounding leaf folding, leading to increased mechanical and photooxidative damage during dehydration. These same features also reduce selective pressure for VDT by providing mechanisms for water retention and dehydration avoidance. Despite the anatomical constraints experienced by vascularized sporophytes, VDT is observed in the sporophytes of some pteridophytes and angiosperms (discussed in detail below), as exemplified by nascent model clades *Selaginella* and *Pleopeltis*. These systems suggest that specific anatomical predispositions such as small stature, narrow vascular elements, flexible cell walls, and thick leaves (Figure 3) may facilitate VDT (Sherwin et al., 1998; Moore et al., 2006). Filmy ferns offer an interesting case study because their sporophytes are anatomically similar to gametophytes, with features such as thin laminae (often just one cell thick), minimal cuticles, and sparse stomatal distribution (Aros‐Mualin and Kessler, 2024). Many filmy ferns have sporophytic DT, indicating that the shared anatomy of filmy ferns and gametophytes may lower the structural barriers to VDT and/or increase selective pressure for VDT, ultimately favoring either the retention and/or the reemergence of VDT (Shah et al., 2019; Nitta et al., 2021). Similar to other highly convergent syndromes, we speculate that assembling the correct anatomies, or anatomical modules, for VDT may be more limiting than evolving the necessary metabolic and biochemical adaptations (Edwards, 2019).

**Figure 4:**
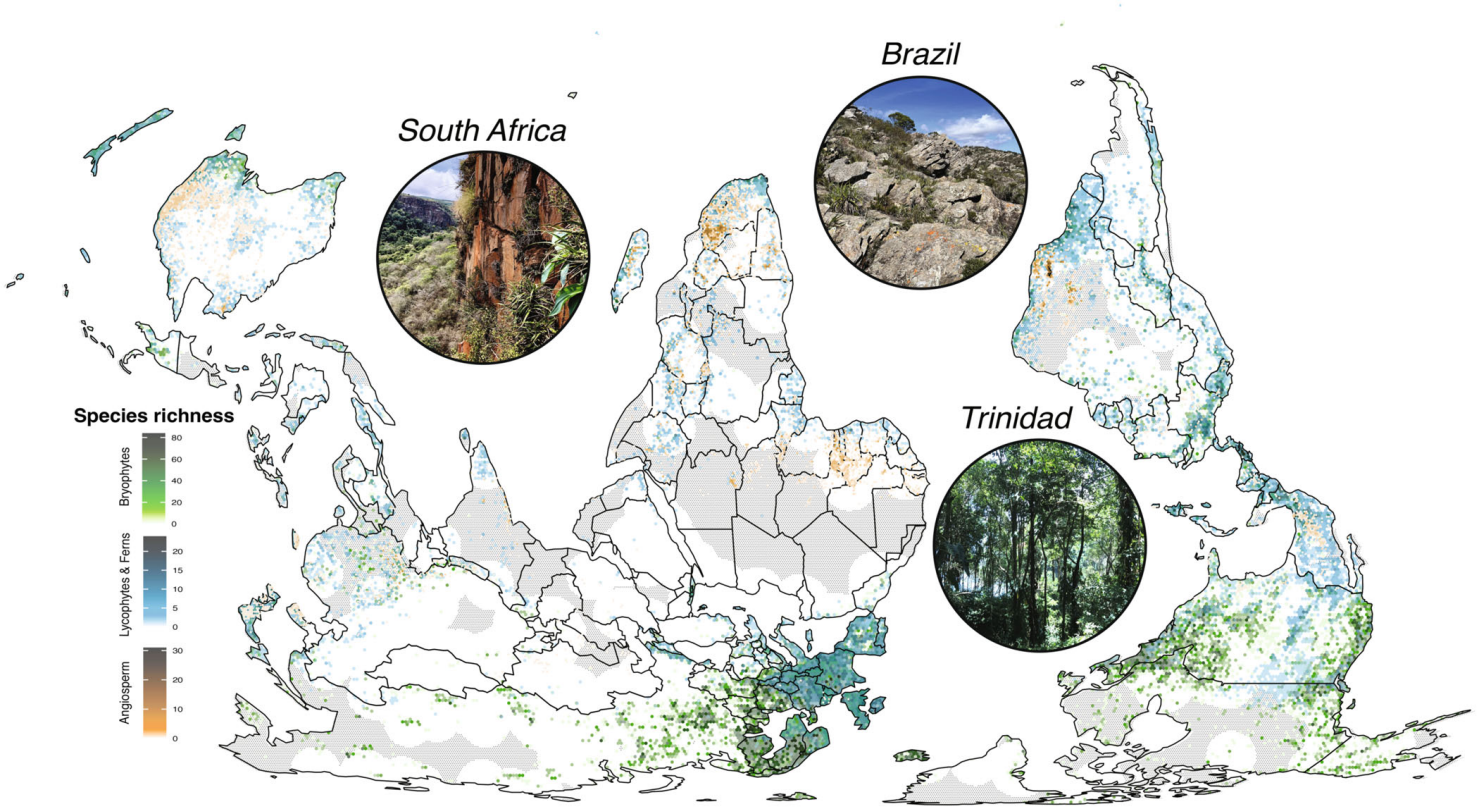
Species richness of plants with vegetative desiccation tolerance (VDT). The local richness of VDT bryophytes, pteridophytes, and angiosperms was calculated from our curated list of confirmed VDT plants (Appendix S1). Diagonal hashes show data‐poor regions of the globe with respect to tracheophyte diversity calculated based on the ignorance map (Rocchini et al., 2011) from Sabatini et al. (2022), which represents areas of the globe more than 500 km from the 1.12 million global vascular plant survey plots. Data gaps for bryophytes are unknown but likely even more extreme. Inset photographs depict characteristic habitats of different types of DT plants in South Africa, Brazil, and Trinidad and Tobago.

We suggest that VDT in sporophytes represents a reestablishment of ancestral, gametophytic pathways, with a convergent twist. Structural bottlenecks likely constrained VDT evolution because lignified cell walls resist deformation, vascular elements cavitate and embolize (Volaire, 2018), and stomata stop exchanging gas when cells shrink during dehydration (Brodribb and Michele Holbrook, 2003). These features prevent the successful integration of core DT molecular modules into sporophytic vegetative tissues until the evolution of specific anatomical predispositions necessary for re‐establishing VDT occurs. This reemergence of VDT depends not just on anatomical compatibility, but also on exposure to the appropriate ecological filtering and selective pressures (Bondi et al., 2023). Support for this interpretation comes from the observation that most VDT sporophytes are associated with habitats where moisture availability is unpredictable and limited, such as epiphytic niches, exposed rocks, and drylands more broadly (Figure 4). In these extreme habitats, DT provides an advantage, paving the way for VDT to reemerge in the sporophytes of species restricted to these sites.

Differences in ploidy between gametophytic and sporophytic tissues add another layer of complexity to understanding the expression of DT across life stages and tissues. Genes or regulatory networks that evolved in haploid contexts may need to be modified, re‐regulated, or undergo subfunctionalization to be expressed effectively in diploid tissues (Menand et al., 2007; Pires et al., 2013; Sakakibara et al., 2013). Transcriptome studies show that there is substantial overlap between gametophyte and sporophyte gene expression in both bryophytes and ferns (Szövényi et al., 2011; Frank and Scanlon, 2015). In contrast, seed plants exhibit much greater divergence between life stages, indicating that sporophyte dominance involved extensive remodeling of ancestral gametophytic regulatory networks (Honys and Twell, 2003). Many DT angiosperms are polyploid, and researchers have long speculated that increased polyploidy may facilitate VDT evolution by providing more genetic material for the production of critical end‐point metabolites or neofunctionalization of duplicated gene copies for deployment in vegetative tissues (Goldblatt and Poston, 1988; Alcantara et al., 2015). However, many VDT angiosperms are also diploid, contradicting the hypothesis that increased ploidy facilitates VDT. While a global pattern of ploidy and DT has not been identified, our recent work suggests that increased ploidy can elevate VDT in a common background or single species (Marks et al., 2024a).

The transition to vascularized sporophyte dominance was a pivotal moment in the evolutionary history of land plants broadly, and VDT specifically. Sporophyte dominance simultaneously relaxed the selection for and increased the constraints on VDT. On one hand, improved water transport and dehydration avoidance mechanisms reduced the frequency of severe dehydration, diminishing the selective pressure for VDT. On the other hand, increased tissue complexity and rigidity imposed structural barriers to surviving and recovering from desiccation (Figure 3). These factors coincide with the widespread loss of VDT in vascularized vegetative tissues and may have set the stage for another transition–the retention of DT only in diaspore tissues, which served as a refugium for core DT molecular modules that were subsequently reestablished in vegetative tissues (Figure 2).

## EVOLUTIONARY REFUGIA AND THE DIASPORE DESICCATION‐TOLERANCE (DDT) FUNCTIONAL UNIT

While VDT is absent from the sporophytes of most tracheophytes (Figure 1), DT has been consistently maintained in the diaspores of nearly all land plant lineages (e.g., spores, pollen, and seeds). In fact, seeds are almost universally DT (90% of seeds are orthodox or DT) (Wyse and Dickie, 2017), reflecting their role as long‐distance dispersal and survival units. Pollen grains, especially in angiosperms, are also often DT, particularly in taxa that rely on wind pollination or experience exposure to dry air before reaching the stigma (Franchi et al., 2011; Pacini and Dolferus, 2019). In these diaspore tissues, DT is developmentally programmed and can be coupled with dormancy (Smolikova et al., 2020). When studied comparatively, even in taxa that have lost DDT, these diaspores provide a window into the evolutionary trajectory of DT across land plant lineages.

At the anatomical level, many diaspores possess features that facilitate DT. Unlike vegetative tissues, spores, seeds, and pollen lack extensive vascular systems and are often small and compact in structure (Linkies et al., 2010) (Figure 2). They are usually not photosynthetically active at the time of dispersal, removing constraints related to chloroplast integrity and photooxidative damage during dehydration (although we should note that many seeds do experience noteworthy photooxidative stress during rehydration and germination). Seed coats and spore walls are typically robust and often enriched with protective polymers or lignin‐like compounds, which provide mechanical stability and reduce the rate of water loss (Huss and Gierlinger, 2021). Together, the low metabolic activity and small, compact cellular architecture of these structures support survival in the dry state by reducing the likelihood of structural and photooxidative damage during desiccation.

The high frequency of DDT is also shaped by strong selective pressures. Inherent in the sessile nature of plants, dispersal tissues are under intense viability selection in environments where water availability is heterogeneous (Beckman and Sullivan, 2023). In fact, this selection pressure may have driven the expansion and ubiquity of seed plants following the dry down associated with the formation of Pangea (Parrish, 1993). In arid environments, desiccation is an inevitable part of the reproductive cycle and selection to ensure propagule longevity is high. Even in systems where moisture is more consistent, DDT is often retained, possibly due to the unpredictability of dispersal and establishment. However, in highly stable and wet environments such as lowland tropical rainforests, some species have evolved recalcitrant seeds that do not tolerate drying and must germinate rapidly to survive (Berjak and Pammenter, 2013). Similarly, in environments where the window for successful pollination is brief, such as in humid, tropical regions or in aquatic water‐pollinated systems, recalcitrant pollen may be advantageous (Franchi et al., 2011). In the case of chlorophyllous (green) spores and seeds, the selective pressure to maintain DT in the diaspore life stage remains high, but the length of DT viability can become greatly reduced in the absence of a photoprotective module (López‐Pozo et al., 2018). Species that have effectively established VDT in their spores have presumably co‐opted or convergently evolved a photoprotective molecular module and may offer a unique window into the evolutionary dynamics of DT. These examples of recalcitrant diaspores represent evolutionary modifications of DT that highlight the power of selection on DT maintenance.

**Figure 5:**
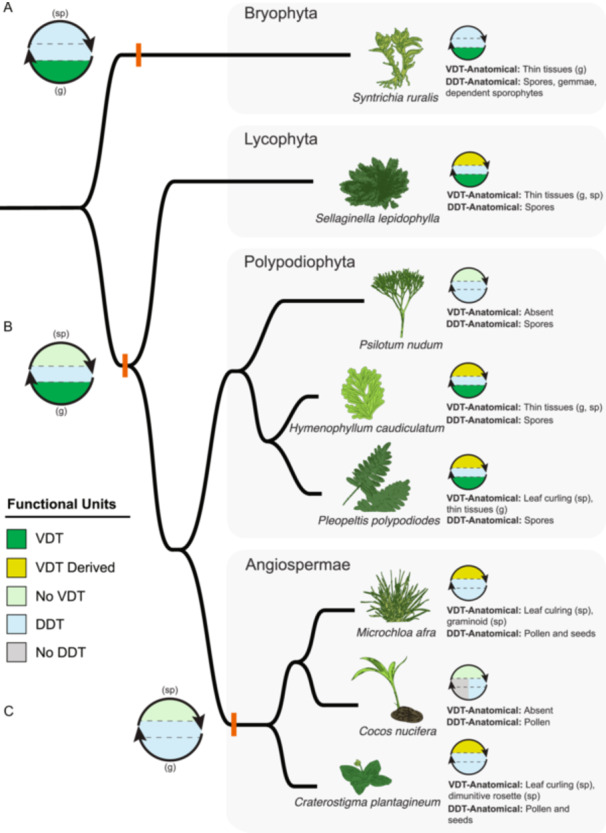
Major evolutionary trajectories of desiccation tolerance (DT) across land plants (Embryophyta). (A) The hypothesized ancestral condition of bryophytes and potentially all land plants is characterized by vegetative desiccation tolerance (VDT) in the gametophyte and diaspore DT (DDT) in the spores and sporophyte. (B) The hypothetical ancestor to vascular plants shows a shift where the sporophyte loses DDT, while the gametophyte retains VDT, with DDT remaining in the spores, a pattern seen in lycophytes. At the origin of angiosperms (C), VDT is absent in the ancestor, and DDT is extended to encompass the seed and pollen, which are a combination of the gametophytic and sporophytic life stages. Across all major lineages shown, the evolution of DT is characterized by the presence, variability, and specific anatomical modules required for the expression of VDT and DDT in different life stages.

We emphasize that DDT has served as an evolutionary refugium for the core protective and reparative molecular modules of DT, preserving critical genetic architecture throughout land plant evolution. In this model, shifting environmental pressures have reshaped the adaptive landscape of functional DT modules, selecting against VDT but for DDT in many tracheophytes (Figure 4). Where VDT thrives in conditions of sporadic wet–dry cycles, DDT is nearly always conserved, providing a “refugium” for the underlying core molecular modules. The maintenance of DT modules in the diaspore refugia ensured that many of the genes and pathways associated with DT (e.g., carbohydrate metabolism, IDPs, and various antioxidants) remained under purifying selection even when they were not expressed in vegetative tissues. These core molecular modules of DT provided the necessary substrate for later convergent reestablishment of VDT given the necessary anatomical predispositions and ecological context (Figure 5).

## REESTABLISHMENT OF VEGETATIVE DESICCATION TOLERANCE IN VASCULAR SPOROPHYTES

The punctuated distribution of VDT across land plants suggests that it is both ancient and recurrent. Like other highly convergent complex traits (e.g., C_4_ and CAM photosynthesis), reestablishing DT in vegetative tissues is not just a simple matter of altering gene expression (Edwards, 2019). It is contingent on overcoming several biological constraints, including structural incompatibilities and physiological limitations, as well as establishing critical protection of the photosynthetic apparatus. Interestingly, different angiosperm clades have followed strikingly different evolutionary trajectories to VDT (Figure 5), each shaped by the interaction of three key factors: anatomical predispositions that enable VDT re‐establishment, ecological opportunities that favor DT selection, and phylogenetic constraints that limit evolutionary flexibility. These diverse trajectories reveal fundamental principles about how complex traits navigate evolutionary landscapes.

The grasses (Poaceae) exemplify how anatomical predispositions can enable repeated evolutionary transitions. At least 45 species across 12 genera have evolved VDT. This appears to have happened independently and recurrently, transitioning multiple times even within single genera such as *Eragrostis* and *Sporobolus* (Marks et al., 2021a, 2024b). These transitions have occurred exclusively in the chloridoid subfamily of grasses and are consistently associated with xeric habitat occupancy (Figure 4). Critically, the anatomical functional module for VDT was already in place in many of these grasses: small size, graminoid growth forms, narrow leaves, high antioxidant potential, and water‐conservative C_4_ physiology (Pardo and VanBuren, 2021) (Figure 3). These predispositions likely facilitated the colonization of xeric habitats where selective pressure for DT was high, creating multiple evolutionary opportunities for VDT reestablishment.

The Velloziaceae family presents a contrasting trajectory that illustrates how ecological restriction can promote trait retention. Unlike the repeated transitions in grasses, VDT appears to have evolved just once in Velloziaceae, after which it was retained across a large species radiation (Gaff, 1987; Alcantara et al., 2018; Porembski et al., 2021) (Figure 1). Species in Velloziaceae share many anatomical features with VDT grasses—graminoid growth forms, narrow leaves, high antioxidant potential—but occupy a much narrower ecological niche on high‐exposure rock outcrops (Alcantara et al., 2018; S. Teodoro et al., 2021). Many species are endemic to very narrow geographical ranges with putatively low effective population sizes and genetic diversity (Bugado et al., 2025). The combination of ecological specialization and extreme selective pressures may explain why VDT, once evolved, was retained across the entire clade.

Across eudicot lineages, the sparse occurrence of VDT (~35 species in five distantly related families; Figure 1) reveals the power of both convergent anatomical solutions and phylogenetic constraints (Marks et al., 2021a). Despite their phylogenetic distance, most VDT eudicots have independently evolved remarkably similar anatomical features: small rosette growth forms, low surface‐to‐volume ratios, and high antioxidant potential (Farrant et al., 2007; Fernández‐Marín et al., 2020; du Toit et al., 2021), although notable exceptions do exist, such as the woody resurrection plant, *Myrothamnus flabellifolia* and the sprawling eudicot *Lindernia brevidens*. Like VDT monocots, other angiosperms are often restricted to xeric and exposed habitats (Figure 4). Phylogenetic constraints may also restrict the evolution of VDT in eudicots. This striking convergence is best illustrated by communities of diverse VDT plants spanning nearly 500 million years of evolutionary divergence growing together in tightly intertwined assemblages on rock outcrops across the arid tropics (Porembski, 2011). These communities provide a powerful demonstration of how ecological filtering can direct evolution toward similar solutions (Figure 4).

These alternate trajectories reveal that VDT evolution is governed by predictable interactions between anatomical possibility and ecological opportunity. Anatomical predispositions do not directly enable VDT reestablishment; they enable colonization of habitats where selection for VDT is strong, creating the ecological backdrop necessary for evolutionary transitions. The frequency of VDT transitions also appears related to ecological breadth: broadly distributed groups like chloridoid grasses show multiple independent transitions, while ecologically restricted groups like Velloziaceae show single origins followed by radiation. Together, these patterns suggest that the evolutionary trajectories of VDT are shaped by the interplay between intrinsic organismal constraints and extrinsic ecological pressures, with anatomical predispositions serving as evolutionary stepping stones that mediate which lineages can access VDT.

## EVOLUTIONARY TRAJECTORIES, CO‐OPTION, AND CONVERGENT EVOLUTION

The “re‐evolution” of VDT in sporophytes presents an evolutionary puzzle. Although VDT has arisen independently in multiple tracheophyte lineages, including nascent model clades Poaceae, Linderniaceae, and Velloziaceae, it remains remarkably rare despite the clear survival advantages it can confer. If the molecular mechanisms for DT already exist in seeds and other diaspores, why do we not see more sporophytic tissues simply reactivating these pathways? The rarity of VDT reestablishment suggests that simply turning ancestral mechanisms “back on” is insufficient or that there are costs associated with VDT.

We propose that core DT molecular modules persist through evolutionary transitions much like species that survive environmental catastrophes in climatic refugia (Figure 2). Just as species retreat to suitable refugia during environmental stress, core DT molecular mechanisms “retreated to” and were preserved within the DDT functional unit, where they remained under purifying selection and avoided evolutionary loss. This refugia model helps explain why core DT modules cannot easily reestablish in vegetative tissues even under favorable conditions. Just like species emerging from refugia, these molecular modules require specific conditions before they can expand into new functional contexts.

Genomic evidence supports this model of a DDT refugium. Comparative work shows that VDT reestablishment is accompanied by expression of core stress‐associated gene families—LEAs, HSPs, and other IDPs—that are also central to DDT modules (VanBuren et al., 2017; Artur and Kajala, 2021). These core DDT modules are then supplemented with expanded photoprotective modules essential for VDT. The gene regulatory networks of these underlying DDT protective and reparative modules show more widespread similarity across species than the convergent photoprotective modules, reflecting their common origins in DDT rather than independent or convergent reestablishment (Illing et al., 2005; VanBuren et al., 2017; VanBuren, 2017; Costa et al., 2017a). Such regulatory conservation suggests that many ancestral networks, preserved in DDT refugia, were subsequently supplemented for the new developmental and environmental contexts of VDT rather than evolving de novo.

However, successful deployment of core DT modules in vegetative tissues requires two critical prerequisites that help to explain VDT's evolutionary constraints. First, lineages must evolve appropriate anatomical predispositions. Plants with VDT often simplify their structure, a trend seen in the one‐cell‐thick leaves of bryophytes and filmy ferns (Aros‐Mualin and Kessler, 2024) or the compact graminoid morphology of many angiosperm resurrection plants (Porembski et al., 2021). These anatomical features likely represent evolutionary “stepping stones” that reduce structural incompatibilities and facilitate the reestablishment of VDT. Second, VDT often requires acquisition of expanded photoprotective molecular modules that supplement core DDT mechanisms (although VDT in roots and nonphotosynthetic tissue is an exception that should be explored in future work). These photoprotective components, including early light inducible proteins (ELIPs), pigment synthesis pathways, nonphotochemical quenching, and poikilochlorophylly, exhibit the hallmarks of convergent evolution (Beckett et al., 2000; Farrant et al., 2007; Challabathula et al., 2018; VanBuren et al., 2019; Alejo‐Jacuinde et al., 2020; Rudresh et al., 2023). Importantly, these modules are rarely expressed in drying seeds nor enriched in orthodox seed genomes, reinforcing the idea that VDT represents a unique combination of refugial core mechanisms supplemented with novel and convergently evolved photoprotective innovations.

Our model organizes DT into a hierarchy that helps explain its evolutionary trajectory. Functional units represent ecological units of selection, while constituent molecular and anatomical modules represent specific gene regulatory networks with direct evolvability. This framework builds on established theoretical models from evolutionary developmental biology (Gerhart and Kirschner, 2007) and systems biology (Guimerà and Nunes Amaral, 2005) that emphasize trait compartmentalization and modularity to provide a mechanistic explanation for the evolutionary constraints governing VDT across different tissue types and life stages.

This refugial pattern reflects broader evolutionary dynamics in which complex traits persist through developmental or ecological refugia before reestablishing in other contexts when conditions favor their expression. Examples from other systems highlight that complex traits can persist in cryptic or developmentally constrained stages and reappear when ecological or anatomical contexts favor their expression. Such examples of trait refugia and reestablishment provide useful parallels for understanding the evolutionary trajectories of VDT. In vascular plants, secondary growth (i.e., woodiness) has repeatedly re‐evolved in lineages that have lost woodiness ancestrally (Carlquist, 2013), and compound leaves have reemerged in groups where they were previously reduced to simple forms (Champagne et al., 2007). Similar dynamics are also evident in animal lineages. For example, ancestral traits like insect wings (La Greca, 1980) and vertebrate teeth (Davit‐Béal et al., 2009) have been lost and reestablished multiple times, and traits restricted to larval stages in insects can sometimes be recruited into adult morphologies through changes in regulatory architecture. These analogies help illustrate a broader evolutionary hypothesis: Complex traits can persist in developmentally constrained stages and then reestablish given the right anatomical and ecological contexts.

## CONCLUSIONS

We have laid out a general framework for understanding the evolution of VDT in land plants. Desiccation tolerance was likely present in the gametophytic tissues of early land plants and was retained in diaspores of many species throughout plant evolution. As tracheophytes transitioned to sporophyte dominance, widespread loss of VDT occurred due to changes in anatomy that shifted the ecology of the species. However, the underlying core molecular capacity for DT persisted in diaspores—maintained under purifying selection—and was subsequently redeployed in vegetative tissues of sporophytes given the appropriate anatomical and ecological contexts. This evolutionary hypothesis posits that derived instances of VDT reflect convergent re‐establishment of a shared ancestral toolkit. We therefore argue that the evolution of VDT is best understood not as a binary gain or loss, but as a conditionally expressed, developmentally constrained, and ecologically modulated trait.

Important questions remain about the evolutionary dynamics and functional constraints of DT. How widespread is VDT in different lineages, and to what extent has it been undersampled? What genomic features distinguish lineages that can redeploy VDT from those that cannot? What are the evolutionary trade‐offs between VDT and other traits, such as growth, metabolic rate, and reproduction? To address these questions, future research should integrate trait‐based surveys, phylogenomic analyses, and detailed ecological characterization across a wider range of taxa. Identifying nascent model systems will be useful for disentangling the molecular and developmental underpinnings of DT, but comprehensive surveys of diverse VDT organisms and tissues would truly revolutionize our understanding of DT evolution and ecology. Dessication tolerance provides a striking example of how ancestral traits can persist in developmental refugia and be convergently repurposed to fuel adaptation to extreme environments.

## AUTHOR CONTRIBUTIONS

R.A.M., R.S.A., and J.T.B.E. all contributed equally to the conceptualization, artwork, and writing of this paper.

## Supporting information


**Appendix S1.** DT species table.

## Data Availability

A curated list of desiccation‐tolerant plants is provided as Appendix S1.
